# Food systems transformations, ultra-processed food markets and the nutrition transition in Asia

**DOI:** 10.1186/s12992-016-0223-3

**Published:** 2016-12-03

**Authors:** Phillip Baker, Sharon Friel

**Affiliations:** 1 Health, Equity and Governance Group, School of Regulation and Global Governance (RegNet), Coombs Extension Building, Australian National University, Canberra, Australia; 2 Menzies Centre for Health Policy, Australian National University, Canberra, Australia

**Keywords:** Transnational food and beverage corporations, Foreign investment, Market power, Processed foods, Food systems, Supermarkets, Nutrition transition, Asia

## Abstract

**Background:**

Attracted by their high economic growth rates, young and growing populations, and increasingly open markets, transnational food and beverage corporations (TFBCs) are targeting Asian markets with vigour. Simultaneously the consumption of ultra-processed foods high in fat, salt and glycaemic load is increasing in the region. Evidence demonstrates that TFBCs can leverage their market power to shape food systems in ways that alter the availability, price, nutritional quality, desirability and ultimately consumption of such foods. This paper describes recent changes in Asian food systems driven by TFBCs in the retail, manufacturing and food service sectors and considers the implications for population nutrition.

**Method:**

Market data for each sector was sourced from Euromonitor International for four lower-middle income, three upper-middle income and five high-income Asian countries. Descriptive statistics were used to describe trends in ultra-processed food consumption (2000–2013), packaged food retail distribution channels (1999–2013), ‘market transnationalization’ defined as the market share held by TFBCs relative to domestic firms (2004–2013), and ‘market concentration’ defined as the market share and thus market power held by the four leading firms (2004–2013) in each market.

**Results:**

Ultra-processed food sales has increased rapidly in most middle-income countries. Carbonated soft drinks was the leading product category, in which Coca-Cola and PepsiCo had a regional oligopoly. Supermarkets, hypermarkets and convenience stores were becoming increasingly dominant as distribution channels for packaged foods throughout the region. Market concentration was increasing in the grocery retail sector in all countries. Food service sales are increasing in all countries led by McDonalds and Yum! Brands. However, in all three sectors TFBCs face strong competition from Asian firms.

**Conclusions:**

Overall, the findings suggest that market forces are likely to be significant but variable drivers of Asia’s nutrition transition. The carbonated soft drink market is the most highly concentrated and likely to be most harmful to population nutrition. The grocery retail sector is, in terms of increasing market concentration and thus market power, likely to be the most important driver of ongoing food systems change and ultra-processed food sales in the region. Given it's rapid growth, the food service sector will also contribute significantly to ongoing dietary change.

**Electronic supplementary material:**

The online version of this article (doi:10.1186/s12992-016-0223-3) contains supplementary material, which is available to authorized users.

## Background

This paper describes recent changes in Asian food systems as driven by transnational food and beverage corporations (TFBCs) in the retail, manufacturing and fast food sectors. The aim is to understand the risks for population nutrition by way of the implications for ultra-processed food sales in the region.

Ultra-processed foods are defined as “industrial formulations made entirely or mostly from substances extracted from foods (e.g. oils, fats, sugar, starch, and proteins), derived from food constituents (e.g. hydrogenated fats and modified starch), or synthesised in laboratories from food substrates or other organic sources (e.g. flavour enhancers, colours, and several food additives used to make the product hyper-palatable)” ([1], p40). While fresh foods are typically prepared and eaten in the home at regular times, ultra-processed foods are designed for consumption anywhere at any time and marketed as ready-to-eat or ready-to-heat ‘convenience foods’ for time-pressured consumers [2]. Because they tend to be higher in fat, salt and glycaemic load than unprocessed or minimally processed foods, and because they are deliberately designed, manufactured and distributed in ways that promote their consumption, these foods are associated with rising rates of obesity and diet-related non-communicable diseases (NCDs) globally [3–5].

Recent studies demonstrate that ultra-processed foods including confectionary, savoury snacks, processed meats and soft drinks now dominate the food systems of high-income countries. For example, almost two thirds (61%) of energy in purchases by households in the United States comes from such foods [6]. Ultra-processed foods are becoming increasingly prominent in the diets of populations in middle-income countries [7–9]. Consumption of such foods is rapidly increasing in several middle-income countries of Asia [10, 11].

Such global dietary changes are consistent with a theory of ‘nutrition transition’ and can be partly explained by rising incomes, changing labour market structures and increasing urbanization – factors that generate demand for convenience foods over the course of economic development [12–15]. From a supply perspective, dietary dependency theory proposes that the globalization of food systems results in developing country populations becoming increasingly exposed to and sometimes dependent on the investments and imports of transnational food and beverage corporations (TFBCs) [8, 16]. Through their considerable market power TFBCs can shape global and local food systems in ways that alter the availability, price, nutritional quality, desirability and ultimately consumption of ultra-processed foods and are therefore increasingly implicated as a key nutrition transition driver [8, 9, 17–19].

### The expansion of TFBCs into Asia

There are strong incentives for TFBCs to expand transnationally including their large market capitalizations and profits (providing cash to grow), global brand recognition, knowledge capital (intellectual property, organizational practices, manufacturing and logistical technologies), and their adaptability to local cultures and regulatory contexts [20, 21]. TFBCs in these sectors are increasingly targeting developing Asian country markets for expansion [8, 9]. Although some TFBCs have had a presence in Asia for over a century (e.g. Coca-Cola entered the Philippines in 1912), many domestic markets have been targeted with vigour since the 1980s given their high economic growth rates, rapidly urbanizing lifestyles, young and growing populations, and the adoption of export-led growth strategies favourable to foreign investment [21, 22]. Trade and investment liberalization in Asia has also been extensive over recent decades thereby reducing barriers to the movement of investments, technologies, production capacity, raw materials and final products across borders, allowing TFBCs to more easily penetrate these markets [22–25].

### Defining the food system and sectors of interest

Food systems encompass the pathways by which food travels from farm to fork including growing, harvesting, processing, packaging, transporting, marketing, consuming and disposing of food. Food systems also include the various inputs (e.g. labour, technologies and materials) and outputs generated at each stage. The supply-side sectors of the food system influence the availability, accessibility, affordability and desirability of foods consumed. Demand-side factors shaping consumption include tastes and cultural preferences, demographics, location and knowledge [26, 27].

Traditional and modern food systems coexist and evolve as countries become richer and more urbanized. Traditional food systems are typically characterised by ‘short’ supply chains involving localised production, distribution and consumption of unprocessed and minimally processed staple foods. Modern food systems, in contrast, are characterised by complex, globalised networks of many actors involved various stages of ‘long’ supply chains, orientated towards maximising efficiency so as to reduce costs and increase production of a wider variety of food types. The shift from traditional to modern food systems has been well described in the literature and is closely intertwined with the nutrition transition [27–29].

This paper focuses on transformations in three food system sectors that are highly relevant to the nutrition transition in Asia. First is the grocery retail sector – the ‘supermarketization’ of developing countries has been implicated as an important nutrition transition driver and transnational grocery retailers the most powerful TFBCs transforming food systems globally [30, 31]. Wal-Mart, Tesco and Carrefour, the world’s largest by turnover, had combined sales revenues of US$550 billion (at retail sales prices) equivalent to 9.2% of global grocery retail sales in 2013 [32]. Wal-Mart alone accounted for 6% of global sales. The company has expanded from 4,189 mostly US-based stores in 2001 to 11,020 stores across 27 countries in 2013 [32, 33].

Second, is the food and beverage manufacturing sector. Transnational food and beverage manufacturers have been among the leading firms of economic globalization. The Coca-Cola Company, for example, began to globalize its operations in the early 1900s and today sells its products in more than 200 countries. Nestle a company founded almost 150 years ago was, as of 2013, selling its products in 194 countries with 468 factories operating in 86 countries [33]. Coca-Cola, Nestle and PepsiCo, the world’s three largest, had combined sales revenues of US$311 billion in 2013 (at retail sales prices) [32].

Third, is the food service sector which underwent rapid global expansion in the 1990s. McDonalds, Yum! Brands (KFC, Pizza Hut), Doctor’s Associates (Subway) and Burger King Worldwide are the world’s largest, accounting for US$153 billion or 25% of global sales in 2012. McDonalds accounted for US$87.6 billion or nearly 60% of this, equivalent to 14.4% of global market share [32]. By 2011, 60% of McDonald’s global sales were from outside the US, while for KFC this figure was 80%. In 2012 McDonalds had more than 35,000 outlets operating in over 100 countries, collectively serving more than 70 million customers daily [34]. Yum! Brands had 39,000 outlets worldwide the same year [35].

### Mechanisms

This paper explores the role of two mechanisms hypothesized to link TFBCs in these sectors to food systems change and ultra-processed food consumption. The first is when TFBCs enter new markets, thereby acting as vectors for the spread of ultra-processed foods across borders – a process of ‘transnationalisation’. The extent to which a given country market is or has been transnationalised is discernible by the market share attributable to TFBCs relative to domestic firms [8–10]. Foreign direct investment (FDI) is a significant strategy used by TFBCs to penetrate new markets and achieve market share through acquiring complete or partial ownership of local companies or establishing new wholly-owned subsidiaries [13, 18, 36, 37]. A number of studies demonstrate associations between FDI, ultra-processed food consumption and associated NCDs [8, 38, 39]. Since the 1980s Asia has been the recipient of more FDI than any other developing region – nearly a quarter of the world’s total in 2011 [40, 41]. The entry of TFBCs into previously unexposed markets is likely to trigger more intensive inter-firm competition, including price competition [18].

The second mechanism is ‘market concentration’ whereby increasing market share and thus market power is held by a declining number of firms, usually occurring in later stages of market development [18, 36, 42]. Today, mergers and acquisitions through FDI are the principal (but not the only means) by which market concentration occurs and through which TFBCs grow [18, 36]. Market concentration increases the buying and selling power of TFBCs allowing them to dictate terms of trade, set prices, and cut costs [18, 19, 43]. Through establishing transnational networks of affiliate firms and contractual suppliers this market power can be ‘vertically coordinated’ across global value chains (GVCs) that incorporate all components of the food supply chain including research & development, production, processing, manufacturing, distribution and sales.

The high-income ‘home’ markets of United States and European TFBCs, especially in the processing and retail sectors, are now highly concentrated [19, 21, 30, 44]. Markets are also concentrating regionally as demonstrated in Europe and Latin America, as well as globally [19, 21]. In the manufacturing sector concentration has been highest in ultra-processed market segments, in particular the soft drinks, biscuits, and snack foods categories, rather than the processed food markets as a whole [45, 46]. The dietary implications of increasing market concentration are likely to be context-dependent given that it occurs unevenly and can influence food consumption patterns in ways that are sector and commodity specific [47–49]. The net effect of increasing TFBC power, however, is likely to result in the greater availability and potentially, through reductions in costs, the affordability of ultra-processed foods. Additionally, the intensification of advertising and promotional activities of TFBCs, which is also associated with factors of market power, is likely to result in the increasing desirability of such foods [25, 47].

Yet to date the extent of market transnationalisation and concentration in the region appear to be unknown. In this study we describe recent trends in ultra-processed food sales, changes in grocery retail distribution channels and food service sales in twelve Asian countries. We also analyse changes in transnationalisation and concentration in each sector in the same countries, and in doing so, identify the main TFBCs operating in Asia. Finally, we discuss the implications of these changes for population nutrition.

## Methods

### Countries

We included the following countries for which data were available, categorized by World Bank income bracket: Lower-middle income countries (L-MICs; 2012 gross national income (GNI) per capita US$1,036 to 4,085) included India (IND), Philippines (PHP), Vietnam (VNM), and Indonesia (IDN); Upper-middle income countries (U-MICs; GNI US$4,086 to 12,615) included China (CHN), Malaysia (MYS), and Thailand (THA), and; High-income countries (HICs; GNI > US$12,616) included Australia (AUS), New Zealand (NZL), Singapore (SGP), South Korea (KOR), and Japan (JPN). Australia and New Zealand are included in this analysis because, although technically they are countries of the ‘Asia-Pacific’ region, they are involved in various major trade agreements with Asian nations and we therefore determined it appropriate to characterise their markets.

### Sales and distribution measures

Per capita sales volumes (kg/l per capita) for each product category through retail and food service channels, from 2000–2013 with projections to 2017, were sourced from Euromonitor International Passport Global Market Information Database, 2014 Edition [32]. Various combinations of these categories have been used in analyses of processed food sales by others. As described elsewhere, Euromonitor Passport is not a scholarly database and the data have limitations similar to official government trade and economic statistics [8, 10]. Although this analysis focuses on transnational corporations, the data may underestimate food distribution and sales shares of traditional domestic channels (e.g. via wet markets, non-chain restaurants and street food vendors) because these are not easily captured in formal economic reporting systems. Distribution share (%) of ‘packaged food’ through grocery retail channels data were also sourced from Euromonitor. The term ‘packaged food’ is synonymous with ‘processed food’ in the Euromonitor categorization schema [32]. Euromonitor collects this data from a number of sources including trade associations, industry bodies, business press, company financial reports, company filings, and official government statistics. Sales volume estimates are validated by people working within the food industry. Projections are calculated by establishing a historic market trend and then factoring in likely future industry specific (e.g. regulation) or market specific (e.g. likelihood of recession) changes, and should be interpreted with caution [32].

### Market transnationalisation and concentration measures

We sourced market share data, defined as the percentage of the total industry’s output value represented by each firm, from Euromonitor [32]. These data were used to calculate a ‘transnationalisation’ measure, defined as the percentage of the total sales value of a given country market attributable to firms of foreign origin. Foreign firms were classified as headquartered outside of a given country market and identified through extensive internet searches of company websites and publically available investor databases [50, 51].

The market share data were also used to calculate the four-firm concentration ratio (CR4), a measure of market concentration, for each market and sector. This was defined as the percentage of the total sales value of a given country market represented by the four largest firms in that market. Although there are no established cut-off points for this measure, we adopt the following: a CR4 of less than 40% indicates a competitive market, 50-80% indicates a market oligopoly (when a market is controlled by a small group of firms), and above 80% indicates a highly concentrated oligopoly [47, 52, 53].

### Sectors and product categories

The ‘grocery retail sector’ captures those firms selling predominantly foods and beverages and other everyday groceries and aggregates hypermarkets, supermarkets, discounters, convenience stores, independent small grocers, chained forecourt retailers, independent forecourt retailers, food/drink/tobacco specialists and other grocery retailers as defined by Euromonitor [32]. Because they also sell fast food, some firms are also included in the consumer food service sector.

The ‘manufacturing sector’ captures those firms producing ‘ultra-processed foods’ (see earlier definition) product categories identified previously as contributing most significantly to sugar, salt and fat consumption in Asia: biscuits, confectionary, ready meals, sweet and savoury snacks, and carbonated soft drinks [10]. We also included oils & fats (predominantly vegetable oil; a ‘processed’ but not ‘ultra-processed food’) in this analysis as a significant commodity in recent dietary change in Asia [13, 14].

The ‘food service sector’ captures firms operating cafés/bars, full-service restaurants, fast food, 100% home delivery/takeaway, self-service cafeterias and street stalls/kiosks with 10 or more units in operation in a country (but includes transnational companies with less than 10 in a given country) as defined by Euromonitor [32]. We deliberately broadened this category beyond ‘fast-food’ given that in Asia many fast food companies are adopting a ‘multi-format’ strategy beyond the traditional fixed fast food restaurant [54].

## Results

### Food and beverage manufacturing sector

**Fig. 1:**
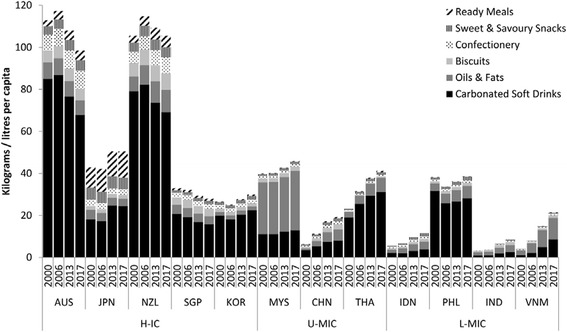
Sales of ultra-processed food products and oils & fats, in selected Asian markets, 2000–2013 with projections to 2017. Footnotes: The product categories were selected as these have been previously identified as contributing most significantly to sugar, salt and fat consumption from ultra-processed foods in Asia; H-IC = high-income countries; U-MIC = upper-middle income countries; L-MIC = lower-middle income countries; see Methods section for other country abbreviations; data from [24]

Figure 1 demonstrates ultra-processed food sales trends. Growth was stagnating or declining in the H-ICs led by reduced carbonated soft drink sales. Sales in Australia and New Zealand far exceeded that of other H-ICs. In the U-MICs and L-MICs sales was increasing in almost all countries, led by growth in the carbonated soft drinks and oils & fats (predominantly edible oil) categories. Of concern was the growing per capita soft drink sales in Thailand, the high volume of soft drink sales in the Philippines, and the high volume of oils & fats sales in Malaysia.

Table 1 describes the sales and market shares of TFBCs operating in this sector at the world, regional and national levels, and the number of countries in which they have operations. Five of the top-ten companies in the food and beverage manufacturing sector were present in every country included in the analysis, suggesting that it was the most ‘transnationalised’ of the three sectors in the region. Although TFBC market share in the processed food sector as a whole was relatively low, it was high in almost all of the ultra-processed food categories we include, in particular the carbonated soft drink and confectionary categories (Additional file 1). Firms of United States and European origin were market leaders. The Coca-Cola Company led the market with more than double the market share of its closest rival PepsiCo. In 2013, 18% of the Coca-Cola Company’s world sales came from the region. Mondelez (formerly Kraft), Mars and Nestle also had a strong trans-regional presence.

**Table 1:** Top ten food and beverage corporations in the Asian ultra-processed food manufacturing sector, 2013, ranked by regional sales

#	Company	Home	World sales (2013 US$B)	Regional sales (2013 US$B)	Regional sales % of world sales	Market share (%)														Number of countries
						World	Region	H-ICs					U-MICs			L-MICs				
								AUS	JPN	NZL	SGP	KOR	MYS	CHN	THA	IDN	PHL	IND	VNM	
1	Coca-Cola Co	US	93.2	16.3	17.5	11.5	9.3	18.9	5.5	13.8	9.8	11.8	5.3	8.7	24.0	11.1	21.4	5.1	5.5	12
2	PepsiCo	US	71.3	7.9	11.1	8.8	4.5	9.2	1.6	7.1	4.2	4.9	3.5	4.5	12.1	2.7	4.8	4.2	6.7	12
3	Wilmar	SGP	7.1	7.1	100.0	0.9	4.0	-	-	-	-	-	-	11.6	-	-	-	-	-	1
4	Mondelez	US	44.5	6.5	14.7	5.5	3.7	10.3	2.2	11.4	12.7	0.1	7.6	2.6	3.6	5.2	2.0	6.1	0.9	12
5	Mars	US	27.4	4.1	15.1	3.4	2.4	6.0	0.1	4.6	3.1	0.8	2.2	4.1	1.3	1.1	1.3	1.0	2.9	12
6	Lotte Group	KOR	3.9	3.6	90.8	0.5	2.0	0.2	3.6	0.1	0.1	23.7	-	0.4	0.7	0.5	-	0.4	2.0	10
7	Nestle	SWT	25.7	3.3	12.9	3.2	1.9	4.5	0.3	3.9	2.3	0.1	1.8	2.9	0.9	1.5	0.4	2.3	0.2	12
8	COFCO	CHN	2.6	2.6	100.0	0.3	1.5	-	-	-	0.9	-	-	4.2	-	-	-	-	-	2
9	Want Want	CHN	2.5	2.4	98.4	0.3	1.4	-	-	-	4.0	-	-	0.3	-	-	-	-	-	2
10	Asahi Group	JPN	2.2	2.2	98.7	0.3	1.3	3.4	3.3	-	-	-	0.3	-	-	-	-	-	-	3
	*Total*		*280.4*	*56.0*		*34.7*	*32.0*	*52.5*	*16.6*	*40.9*	*37.1*	*41.4*	*20.7*	*39.3*	*42.6*	*22.1*	*29.9*	*19.1*	*18.2*	*78*

Footnotes: Market share is combined for the following product categories = biscuits, confectionary, fats & oils, ready meals, sweet and savoury snacks, and carbonated soft drinks, *COFCO* China National Cereals, Oils and Foodstuffs Corporation, *H-IC* high-income countries, *U-MIC* upper-middle income countries, *L-MIC* lower-middle income countries, *SWT* Switzerland, see Methods section for other country abbreviations; data from [24]

Table 2 describes the market concentration for each product category in 2013 and change since 2004. The carbonated soft drink market was the most highly concentrated in all countries with two US firms, Coca-Cola and PepsiCo, having an effective oligopoly. The market share held by market leader Coca-Cola was approaching monopolistic levels in New Zealand (74.3%), the Philippines (74.1%) and Indonesia (89.9%). The confectionary, biscuits, and oils & fats categories were also highly concentrated in almost all countries with the exception of Japan. In Japan, Singapore, China and India there was strong competition with few firms holding a dominant market position. In China, although domestic firms appear to have out-competed foreign rivals in some categories, TFBCs dominated the ultra-processed categories included in this analysis.

**Table 2:** Market concentration in ultra-processed food categories in selected Asian markets, 2013 and change (%) since 2004

	Country	Biscuits		Carbonat. soft drinks		Confectionary		Oils & Fats		Ready Meals		Sweet & Sav. Snacks		Packaged Food	
		CR4	2004-13 ∆ (%)	CR4	2004-13 ∆ (%)	CR4	2004-13 ∆ (%)	CR4	2004-13 ∆ (%)	CR4	2004-13 ∆ (%)	CR4	2004-13 ∆ (%)	CR4	2004-13 ∆ (%)
H-IC	AUS	86.3	11.6	92.6	0.5	72.8	6.9	19.7	9.4	55.2	39.6	59.6	11.8	72.1	0.1
	JPN	46.2	3.4	78.5	8.6	44.6	−2.2	19.5	9.9	58.2	11.8	81.6	0.3	41.4	24.8
	KOR	92.8	2.7	96.8	6.0	66.4	−8.7	25.8	4.5	78.9	8.8	72.0	11.7	62.8	−6.4
	NZL	87.0	0.6	90.0	1.2	76.4	13.0	40.7	17.9	81.7	62.8	48.0	3.5	63.6	1.5
	SGP	66.0	13.6	95.4	5.6	42.1	21.7	22.3	2.3	61.0	5.2	54.8	−1.4	39.8	8.4
U-MIC	CHN	30.2	23.3	95.3	2.4	31.4	139.7	20.9	34.8	71.0	13.4	70.1	85.5	29.4	57.2
	MYS	60.3	22.3	95.2	3.3	52.0	30.7	24.0	−2.0	45.2	−9.4	64.1	2.0	38.3	11.3
	THA	62.3	9.9	91.9	−3.4	50.6	26.2	17.9	2.3	56.7	−0.9	73.7	12.5	48.5	1.9
L-MIC	IDN	61.8	36.4	97.9	4.0	54.1	30.4	62.8	9.2	85.8	5.4	91.5	2.3	46.9	10.9
	IND	85.4	6.1	96.4	−1.1	71.6	9.8	25.0	−3.1	42.3	14.9	71.1	3.9	74.3	−2.0
	PHL	76.4	16.3	98.3	7.5	55.4	31.0	20.7	1.5	76.9	1.1	82.4	6.6	57.5	2.3
	VNM	59.7	24.1	90.3	0.9	61.7	20.7	31.6	6.4	66.4	5.7	70.9	44.7	34.3	14.7
	Average	67.9	14.2	93.2	3.0	56.6	26.6	27.6	7.8	64.9	13.2	70.0	15.3	50.7	10.4

Footnotes: *CR4* four firm concentration ratio, a measure of market power with higher values indicating more concentrated markets, *H-IC* high-income countries, *U-MIC* upper-middle income countries, *L-MIC* lower-middle income countries, *Cabonat.* Caronbated, *Sav.* Savoury, see Methods section for country abbreviations; data from [24]

### Grocery retail sector

**Fig. 2:**
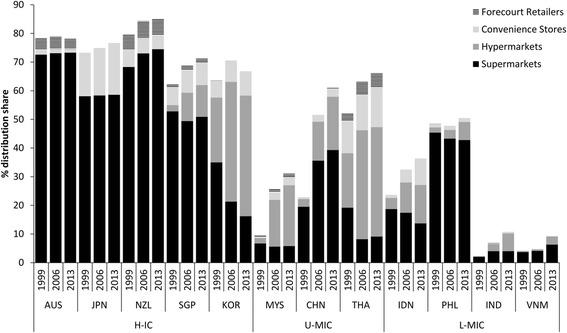
Distribution share (%) of processed foods through modern grocery retail channels, 1999–2013, in selected Asian markets. Footnotes: Remainder of distribution is largely through ‘traditional grocery retailers’; ‘Processed foods’ includes all Euromonitor packaged food product categories; H-IC = high-income countries; U-MIC = upper-middle income countries; L-MIC = lower-middle income countries; see Methods section for other country abbreviations; data from [24]

Figure 2 demonstrates trends in the distribution of processed foods through ‘modern grocery retail’ channels – supermarkets, hypermarkets, forecourt retail and chained convenience stores. Although distribution through these channels had stagnated in H-ICs it was increasing in all U-MICs and L-MICs. Change was most rapid in China with distribution through these channels increasing three-fold from 20% in 1999 to over 60% in 2013. Although only ~10% of processed foods were distributed through these channels in India and Vietnam in 2013, this had more than tripled and doubled respectively since 1999. In some countries forecourt retailers (petrol stations) and chained convenience stores have emerged as important channels although supermarkets and hypermarkets have predominated in all countries.

Table 3 describes the sales and market shares of major TFBCs operating in this sector at the world, regional and national levels, and the number of countries in which they had operations. Of the three sectors, grocery retail demonstrated the highest turnover in sales but the lowest level of transnationalisation. In Thailand TFBC penetration was most evident and increased rapidly between 2004 and 2013 (Additional file 2). Only one firm, Seven & I Holdings (7-Eleven), had a discernible presence across the region, operating in nine countries. Transnationalisation was also highly variable ranging from 5% or less in Australia, Japan and China to negligible values in L-MICs. One of the largest global retailers, the supermarket chain Tesco was among the leading TFBCs in most countries with regional sales representing 18.8% of the companies world sales in 2013. Inter-TFBC retail competition did not appear to be strong, with firms consolidating their operations in key markets – Wal-Mart in Japan, Tesco in Korea and Thailand, and Carrefour in Singapore and Indonesia. These firms and others were, however, competitors in China indicating strong competition. In some markets TFBC retailers have faced strong competition from regional players.

**Table 3:** Top ten food and beverage corporations in Asian grocery retail sector, 2013, ranked by regional sales

#	Company	Home	World sales (2013 US$B)	Regional sales (2013 US$B)	Regional sales % of world sales	Market share (%)														Number of countries
						World	Region	H-ICs					U-MICs			L-MICs				
								AUS	JPN	NZL	SGP	KOR	MYS	CHN	THA	IDN	PHL	IND	VNM	
1	Seven & I	JPN	75.9	57.1	75.3	1.3	3.2	1.6	12.4	-	6.4	4.1	2.9	0.1	13.6	0.1	0.6	-	-	9
2	Woolworths	AUS	43.9	43.9	100.0	0.7	2.5	33.7	-	23.3	-	-	-	-	-	-	-	-	-	2
3	Wesfarmers	AUS	32.9	32.9	100.0	0.6	1.9	28.4	-	-	-	-	-	-	-	-	-	-	-	1
4	CRE	CHN	22.4	22.0	98.3	0.4	1.2	-	-	-	-	-	-	3.3	-	-	-	-	-	1
5	Lawson	JPN	20.8	20.6	99.0	0.3	1.2	-	5.9	-	-	-	-	-	-	-	-	-	-	1
6	Wal-Mart	US	356.1	18.9	5.3	6.0	1.1	-	2.4	-	-	-	-	1.6	-	-	-	-	-	2
7	AEON	JPN	17.7	17.6	99.4	0.3	1.0	-	4.5	-	-	1.1	3.1	-	1.0	-	0.3	-	-	5
8	Tesco	UK	90.2	17.0	18.8	1.5	1.0	-	-	-	-	13.8	7.4	0.4	11.8	-	-	-	-	4
9	Metcash Ltd	AUS	16.7	16.7	100.0	0.3	0.9	14.4	-	-	-	-	-	-	-	-	-	-	-	1
10	FamilyMart	JPN	18.8	16.4	87.5	0.3	0.9	-	4.6	-	-	-	-	-	0.7	-	-	-	-	2
	*Total*		*695.4*	*263.1*		*11.7*	*14.9*	*78.1*	*29.8*	*23.3*	*6.4*	*19.0*	*13.4*	*5.0*	*27.1*	*0.1*	*0.9*	*0.1*	*0.1*	*28*

Footnotes: *CRE* China Resources Enterprise Corporation, *THI* Ting Hsin International, *H-IC* high-income countries, *U-MIC* upper-middle income countries, *L-MIC* lower-middle income countries, see Methods section for other country abbreviations; data from [24]

**Fig. 3:**
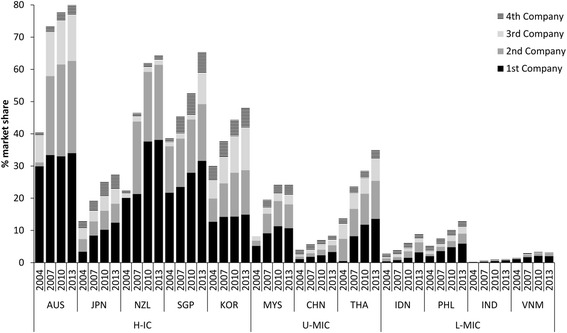
Market concentration in the grocery retail sector (% market share held by leading four grocery retailers), 2004–2013, in selected Asian markets, with company rank indicated. Footnotes: H-IC = high-income countries; U-MIC = upper-middle income countries; L-MIC = lower-middle income countries; see Methods section for other country abbreviations; data from [24]

Concentration in the grocery retail sector varied considerably across the region but had increased rapidly in almost all countries (Fig. 3). Australia, New Zealand and Singapore had CR4 ratios of 79.9%, 64.4% and 65.4% respectively in 2013, indicating strong oligopolistic markets (i.e. weaker competition). Among the emerging economies concentration was increasing most rapidly in Thailand from a CR4 of 13.7% in 2004 to 34.9% in 2013. Relative to countries at similar levels of economic development, Japan and China appeared to have the most competitive markets in the region. Concentration was very low in all L-MICs.

### Food service sector

**Fig. 4:**
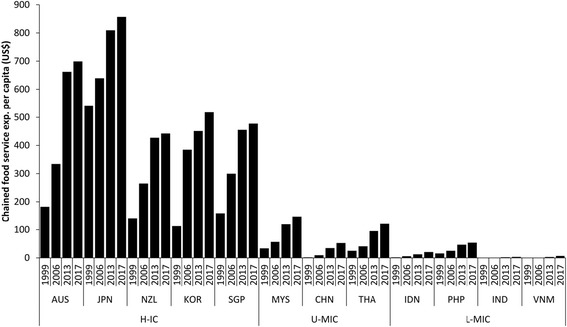
Growth in per capita sales expenditure at food service outlets (US$, fixed 2013 prices and exchange rates), 1999–2013 with projections to 2017, in selected Asian markets. Footnotes: H-IC = high-income countries; U-MIC = upper-middle income countries; L-MIC = lower-middle income countries; see Methods section for other country abbreviations; data from [24]

Figure 4 demonstrates trends in per capita food service sales. Between 1999 and 2013 per capita sales increased rapidly in almost all countries, but predominately in H-ICs. Although in China expenditure per capita increased eighteen-fold from US$1.90 in 1999 to US$34.80 in 2013, expenditure in U-MIC and L-MIC was a small fraction of that in H-ICs. Per capita sales transactions at chained food outlets approximately doubled in Australia and Singapore and tripled in South Korea over the period suggesting that consumers are not only spending more on fast food but also visiting outlets more frequently (Additional file 3).

Table 4 describes the sales and market shares of major TFBCs operating in this sector at the world, regional and national levels, and the number of countries in which they had operations in 2013. There was significant variation in the transnationalisation of this sector across the region. McDonalds and Yum! Brands (Pizza Hut, KFC) were present in all countries (McDonalds only recently entered Vietnam and has little discernible market share) and were market leaders in all countries except Japan and Thailand. Seven & I Holdings (7-Eleven), Starbucks, Doctor’s Associates (Subway), Dunkin Brands Group (Dunkin Donuts), Domino’s Pizza and Burger King Worldwide (Burger King) also had a strong regional presence. The combined market share of Yum! Brands and McDonalds varied from 3.7% in South Korea to 42.5% in Australia. Yet in most countries market share growth by US companies had stagnated with the exceptions of Malaysia, India and Vietnam where it continued to increase.

**Table 4:** Top ten food and beverage corporations in the Asian food service sector, 2013, ranked by regional sales

#	Company	Home	World sales (2013 US$B)	Regional sales (2013 US$B)	Regional sales % of world sales	Market share (%)														Number of countries
						World	Region	H-ICs					U-MICs			L-MICs				
								AUS	JPN	NZL	SGP	KOR	MYS	CHN	THA	IDN	PHL	IND	VNM	
1	McDonald’s	US	89.2	15.3	17.1	13.1	7.4	28.3	5.2	16.1	16.1	2.5	14.5	7.2	2.4	5.5	13.5	11.0	-	11
2	Yum! Brands	US	41.8	14.7	35.2	6.2	7.1	13.5	1.2	13.6	8.4	1.5	21.2	18.7	7.0	22.8	4.9	13.1	45.4	12
3	Seven & I	JPN	18.0	14.4	80.1	2.7	7.0	1.5	12.3	-	3.1	0.7	1.1	-	23.9	0.6	1.5	-	-	8
4	FamilyMart	JPN	4.7	4.4	93.8	0.7	2.1	-	4.3	-	-	-	-	0.1	0.8	-	-	-	-	3
5	Lawson	JPN	4.0	4.0	100.0	0.6	2.0	-	4.0	-	-	-	-	-	-	-	-	-	-	1
6	Zensho	JPN	3.9	3.9	100.0	0.6	1.9	-	3.8	-	-	-	-	-	-	-	-	-	-	1
7	Starbucks	JPN	17.4	3.3	18.7	2.6	1.6	0.3	2.8	-	-	-	-	-	-	-	-	-	-	1
8	Skylark	US	3.2	3.2	99.7	0.5	1.5	-	1.2	1.0	2.2	1.8	2.3	2.4	1.6	3.1	2.6	0.4	0.7	12
9	THI	CHN	2.1	2.1	100.0	0.3	1.0	-	-	-	-	-	-	4.8	-	-	-	-	-	1
10	Uny	JPN	2.1	2.1	100.0	0.3	1.0	-	2.1	-	-	-	-	-	-	-	-	-	-	1
	*Total*		*186.4*	*67.4*		*27.6*	*32.7*	*43.6*	*36.9*	*30.7*	*29.8*	*6.5*	*39.1*	*33.2*	*35.7*	*32.0*	*22.5*	*24.5*	*46.1*	*51*

Footnotes: *THI* Ting Hsin International, *H-IC* high-income countries, *U-MIC* upper-middle income countries, *L-MIC* lower-middle income countries; see Methods section for other country abbreviations; data from [24]

Concentration in this sector also varied considerably across the region (Additional file 4). It was lowest in the H-ICs Japan and South Korea, countries with a higher number of competing domestic firms, and highest in the L-MICs Philippines and Vietnam. Growth in concentration was stagnant or declining in most countries but increasing in Malaysia, Thailand and India.

## Discussion

The results demonstrate that food systems in Asia's middle-income countries are changing rapidly, characterised by increasing ultra-processed food sales, the expansion of modern grocery retail channels, and increasing fast food sales. The manufacturing and food service sectors appear to be highly transnationalised. All sectors are becoming increasingly concentrated, particularly the carbonated soft drink and grocery retail sectors. However there is wide variability between countries as well as between the three sectors. These findings therefore support a theory of dietary convergence-divergence, where market forces associated with globalization are driving a regional convergence in diets towards high levels of ultra-processed food consumption but with divergent consumption patterns at the national level resulting from a range of demographic, cultural, economic, market and policy factors that influence dietary preferences [13, 55]. The changes in Asian food systems that we have described are likely, therefore, to have variable implications for public health nutrition across the region and it is this variability that we now explore.

### Food and beverage manufacturing sector

Existing evidence shows that transnational food and beverage manufacturers have, when entering developing country markets, tended to invest in the ultra-processed food categories in particular soft drinks, snack foods and biscuits [12, 18, 46, 56, 57] the same categories identified as significant sugar, salt and fat vectors in countries across Asia [10]. Our data are consistent with these observations, demonstrating that although TFBC market share in the processed food sector as a whole is relatively low, it is higher in the carbonated soft drink and confectionary categories. Three US firms Coca-Cola Co, PepsiCo and Mondelez are regional leaders. In some categories, such as carbonated soft drinks, they have achieved market shares in L-MICs and U-MICs commensurate with those of H-ICs, and in other categories such as confectionary they continue to expand.

There is also evidence that soft drink companies are, in terms of sales and market capitalization, the largest and most powerful group of TFBCs operating in this sector [47, 55]. Our data support these findings given that market concentration is highest in the carbonated soft drink category across all countries except Japan. Consistent with an earlier study, the results also demonstrate that carbonated soft drink sales is rising rapidly in some countries and is particularly high in Thailand and the Philippines relative to the level of economic development. The actions taken by the Coca-Cola Company and PepsiCo are therefore likely to have important implications for population nutrition in the region. Regulating soft drink markets through the adoption of sales taxes, advertising restrictions, labelling controls and other key interventions will be important determinants of population nutrition [58].

In some countries there is strong competition from Asian players. Lotte Group (Korea), for example, has a presence in ten countries, generating 94% of sales from the region in 2012 [54]. Although Japanese firms are numerically more common in the region, such firms largely serve their home rather than foreign markets through intra-firm supply chains originating from subsidiaries located in countries throughout Asia, in particular China, Thailand and Taiwan [59].

Advertising and promotional activities of TFBCs in this sector is likely to be an important driver of consumer preferences and consumption [60]. In highly competitive markets such as China TFBCs have invested heavily in advertising and promotional campaigns [61]. In Thailand, companies are incorporating not only mass media but also a diversity of social networking platforms, road shows, special events, contests, blogs and celebrity brand ambassadors into their marketing mix [61]. Marketing to children is pervasive in some countries. One survey reported that in India 40-50% of advertisements were for food during children’s programming, in the Philippines 50-75% and in Malaysia 70% [62].

Transnational food and beverage manufacturers have also adopted a ‘glocalization’ strategy to expand sales in the region, the synonymous transnationalisation of brands and products but with adaption to local cultures of consumption and regulatory contexts. To achieve this, manufacturers have invested heavily in research and development (R&D) activities in the region. In China, for example, Mondelez launched a ‘Golden Oreo’ in 2013 designed to appeal to local taste preferences but also the ‘good fortune’ associated with that colour in Chinese culture [61]. Oreo’s now account for 40% of the company’s food sales in China [63].

### Grocery retail sector

In grocery retail but not in the manufacturing and food service sectors our analyses demonstrate that concentration is increasing in almost all countries. Concentration appears to be very low in all L-MICs indicating the potential growth opportunities for retailers. We have also demonstrated that processed food distribution through modern grocery channels is increasing rapidly in U-MICs. These data indicate that the grocery retail sector is, in terms of increasing market concentration and thus market power, likely to be a key driver of ongoing food systems change and consumption patterns in the region. The data supports the observation that the supermarketisation of Asia is not only well underway [12, 64, 65], but is also continuing apace.

Ongoing supermarketisation is likely resulting in some positive outcomes for nutrition, for example the greater availability of safer and more diverse foods [30]. However, supermarkets also establish important distribution channels for ultra-processed foods, and irrespective of food type, may encourage the consumption of more food generally [13, 14, 30]. The nutritional implications are, however, likely to be context specific. Hawkes identifies five factors that shape the nutritional implications of supermarketisation, largely involving the strategic choices taken by supermarket operators including retail location and format, food types sold, pricing, promotion and nutrition-related activities [30]. As the region’s largest and most transnational grocery retailer such strategic choices made by Seven & I (7-Eleven) are likely to be important for population nutrition.

Within countries, supermarkets spread from major cities to intermediate and small towns, reflecting an initial targeting of wealthier middle-class consumer segments before targeting poorer urban and rural segments [36, 65]. Although introducing improved food safety standards, they act predominantly as new distribution channels for durable processed foods in the early stages of market growth, before offering a wider diversity including fresh foods (and out-competing wet markets) in later stages [13, 14, 36, 64]. As a result supermarket market shares in the packaged, processed and dry food categories such as grains, noodles and dairy products have increased much more rapidly than in the fresh food categories [66]. This reflects not only the greater economies of scale associated with sourcing processed food [13, 18], but also because ‘cultures of consumption’ take time to change from the daily purchase of fresh foods in wet markets to the less frequent purchases from modern grocery retailers and refrigerator storage [67, 68].

In the early stages of market penetration transnational grocery retailers can also act as ‘Trojan horses’ for imported processed foods as they link their operations in emerging markets to their more secure global sourcing networks and supply chains (e.g. Wal-Mart was importing 55% of its products into Mexico in 2003, particularly from China) [53, 69]. However, as they invest in local supply-chain improvements, national and regional sourcing networks may become increasingly prominent. This ‘sourcing evolution’ which includes both global, regional and local sourcing, may be most prominent at the regional level in Asia with its deeply integrated markets and advanced cross-border production networks, particularly with the advent of the ASEAN Economic Community in 2015 [69, 70].

Conversely they can also act as ‘export platforms’ whereby processed food products sourced in the new market are exported into the home or other markets in which the firm operates [53]. China, as the world’s largest processed food manufactory by volume, is the regions ‘global sourcing export gateway’, partly explaining why all TFBCs in this sector have a market presence there [53]. The establishment of such intra-firm supply chains may, therefore, represent a key mechanism by which transnational grocery retailers facilitate the regional integration of ultra-processed food markets and consumption.

### Food service sector

We have demonstrated that transnational fast food companies are rapidly penetrating Asian markets and that this sector is growing rapidly in H-ICs and U-MICs, particularly in China. This may have a significant impact on diet-related NCD risks in many countries, given that the availability and price of fast-foods have been associated with obesity at both national and global levels [12, 71, 72].

Two firms, McDonalds and Yum! Brands, are at the forefront of the observed market changes. They have transnationalised through a mixed model of establishing wholly company-owned outlets and entering into franchise licence agreements with local owners or corporate affiliates [73]. McDonalds outlets in the Asia-Pacific region increased from 1,458 (11.7% of total) in 1991 to 6,775 (23.3%) in 2001 [74]. Yum! Brands is the most successful firm in China, benefiting from its early entry and ‘first-mover’ advantage but also because its largest brand KFC is chicken-based, a meat more popular with Chinese consumers than beef [75]. In the most important regional market, China, expansion by these two firms has been rapid. The market leader Yum! Brands established 656 new restaurant outlets in 2011 and 889 new outlets in 2012, bringing its total to more than 5750 operating across 850 cities the same year [35].

It is also apparent that these companies are not only spreading between countries but also within them. In China, for example, Yum! Brands initially expanded rapidly in major (first and second-tier) cities but since 2010 it has added more outlets in smaller (third to sixth-tier) cities than in the former. The company aims to establish 20,000 outlets across China in the long-term [35]. Together these results suggest that the full potential contribution of this sector to the nutrition transition in Asia is only now being realized in the H-ICs and is yet to be realized in the U-MIC and L-MICs. Fast food is likely to become increasingly significant in the diets of lower-tier urban populations.

In the food service sector, franchising is a key strategy for glocalization as it allows firms to acquire local knowledge about consumer preferences, supply-chains and business practices thereby making the brand more competitive [73]. Transnational fast food companies have localized their products to meet the taste preferences of Asian consumers. McDonalds, for example, offers rice porridge with chicken and pork in Thailand, a diversity of vegetarian options in India, rice-based wraps, bowls of chicken and ‘bubble tea’ (a mixture of tea, milk and sweet tapioca balls) in China [74, 76].

Compared with North America, Europe and Australasia where chained operators dominate, TFBCs in this sector face stiff competition in Asia from thousands of small, independent restaurant operators selling local dishes at often very low prices, especially in China and India [76, 77]. These operators tend to serve low-income Asian consumers unable to afford the more expensive foods sold by TFBCs. Many chained operators have responded by offering lower-priced snack-items through small kiosk-type outlets and aggressively marketing to low-income consumers. This strategy is profitable in the long-term as chained operators can achieve brand loyalty from consumers with rising disposable incomes [9, 78]. In all countries except Indonesia street food dominates Southeast Asian food service, comprising (among ASEAN countries) 55% of regional transactions in 2011. Street food takes several forms, ranging from the hawker centres of Singapore that claim 78% of total food service transactions, to the street stalls of Thailand with 74%. In response, chained operators are in some markets adopting a ‘multi-format’ strategy establishing small street kiosks that can compete with street vendors alongside their fixed restaurant outlets [78].

### Limitations

This analysis has a number of limitations. First, TFBC market shares were likely under-estimated as it was not always possible to ascertain foreign vs. domestic ownership. Ownership structures are often highly complex involving multiple shareholders. However the Euromonitor market share data we have used indicates the ‘global brand owner’ which is the ‘ultimate owner’ of brand. Additionally, with the exception of the food service sector Euromonitor does not list firms with < 0.1% market share and the data did not therefore capture the presence of a TFBC in a given country with less than this value. Although we have provided a descriptive analysis of Asian ultra-processed food markets across the three relevant sectors, statistical associations with consumption have not been established and further research is required to this end. While we have sought to situate our analysis within the wider milieu of possible drivers of ultra-processed food consumption and the nutrition transition, the nature of the analysis does not allow for control or inclusion of such variables. This analysis has focused on the role of TFBCs as drivers of nutrition transition in Asia. However, this should not detract from the significant role that small and medium-size food and beverage corporations are also likely to play [79].

## Conclusions

In an increasingly globalised world it is helpful to understand how markets and commercial actors operate in ways that affect public health. Our results suggest that market forces are likely to be important ‘upstream’ drivers of Asia’s nutrition transition and can help to explain variability in ultra-processed food consumption across the region. Consistent with findings from other regions, TFBC market share and market concentration are highest in the carbonated soft drink category. The food service sector is still growing in H-ICs and is growing rapidly in U-MICs with fast food likely to become increasingly significant in the diets of urban populations outside of the major centres. Importantly, ongoing food systems transformations in Asia are, as they relate to ultra-processed food consumption, likely to be driven by ongoing concentration in the grocery retail sector in particular. Although several Asian TFBCs are strong competitors in many markets it is uncertain how characteristically different these corporations are to their US and European counterparts.

## Additional files

Additional file 1: Market share held by foreign firms in selected ultra-processed food categories (%), 2013, in selected Asian markets, with firm origin indicated (PDF 378 kb)
Additional file 2: Market share held by foreign firms in the grocery retail sector (%), 2004–2013, in selected Asian markets, with firm origin indicated (PDF 212 kb)
Additional file 3: Sales transactions per capita at food service outlets, 1999–2013 with projections to 2017, in selected Asian markets (PDF 159 kb)
Additional file 4: Market concentration in the food service sector (% market share held by leading four firms), 2003–2012, in selected Asian markets, with company rank indicated (PDF 205 kb)

## Abbreviations

AUS: Australia
CHN: China
GNI: Gross national income
HICs: High-income countries
IDN: Indonesia
IND: India
JPN: Japan
KOR: South Korea
L-MICs: Lower-middle income countries
MYS: Malaysia
NCDs: Non-communicable diseases
NZL: New Zealand
PHP: Philippines
SGP: Singapore
TFBCs: Transnational food and beverage corporations
THA: Thailand
U-MICs: Upper-middle income countries
VNM: Vietnam
