# Supplementary material for: Food systems transformations, ultra-processed food markets and the nutrition transition in Asia
Source: Global Health. 2016 Dec 3;12:80. doi: 10.1186/s12992-016-0223-3 (PMC5135831; doi:10.1186/s12992-016-0223-3)
Supplement: Additional file 1: — Market share held by foreign firms in selected ultra-processed food categories (%), 2013, in selected Asian markets, with firm origin indicated (PDF 378 kb) [file 12992_2016_223_MOESM1_ESM.pdf]

Additional file 1. Market share held by foreign firms in selected ultra-processed food categories (%), 2013, in selected Asian markets, with firm origin indicated

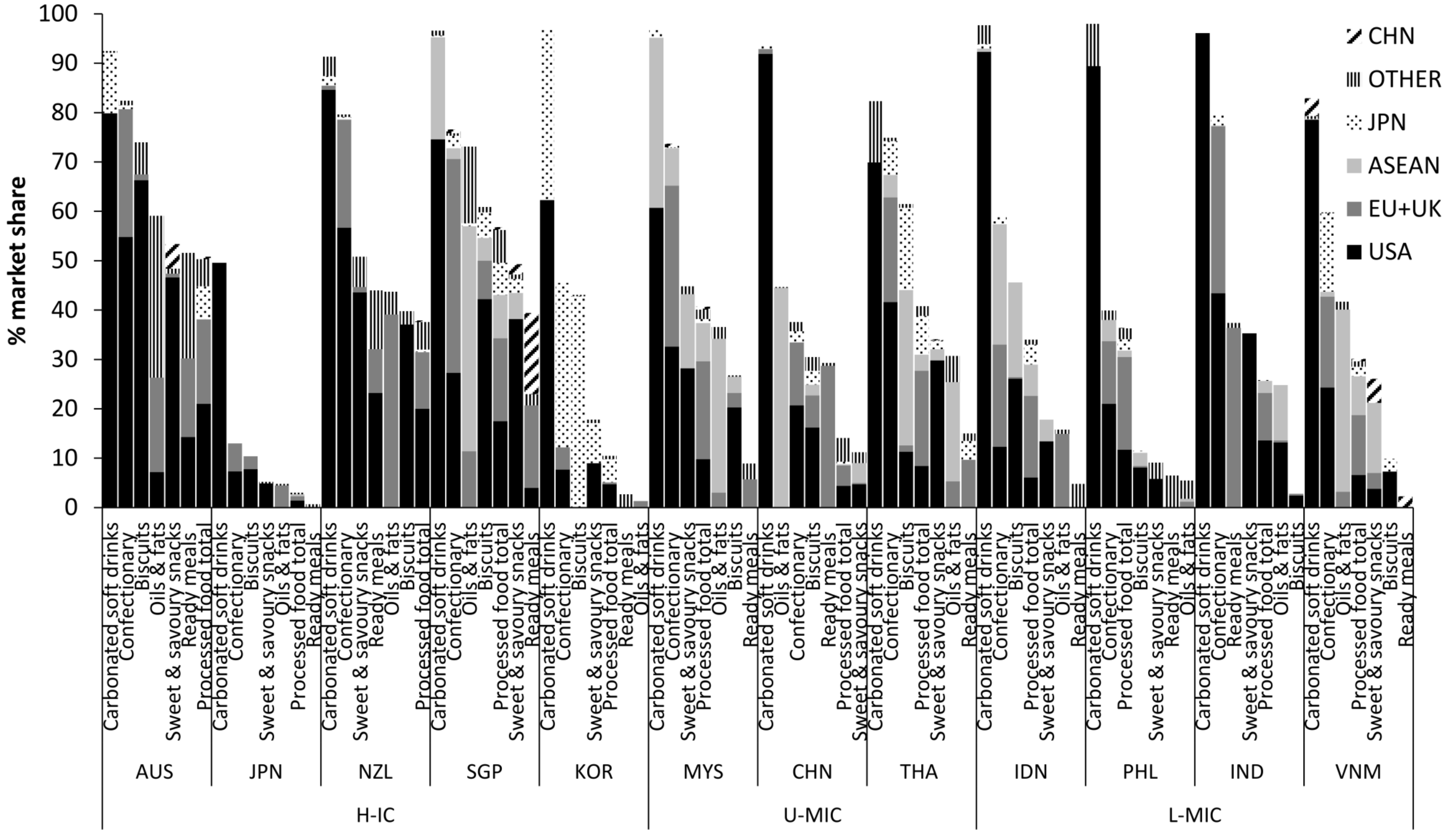

Footnotes: For each country product categories are ranked from highest (left) to lowest (right) total foreign market share; ASEAN = Association of South East Asian Nations; USA = United States of America; EU + UK = European Union and United Kingdom; H-IC = high-income countries; U-MIC = upper-middle income countries; L-MIC = lower-middle income countries; see methods section for other country abbreviations; data from [24].
