# Supplementary material for: Food systems transformations, ultra-processed food markets and the nutrition transition in Asia
Source: Global Health. 2016 Dec 3;12:80. doi: 10.1186/s12992-016-0223-3 (PMC5135831; doi:10.1186/s12992-016-0223-3)
Supplement: Additional file 2: — Market share held by foreign firms in the grocery retail sector (%), 2004–2013, in selected Asian markets, with firm origin indicated (PDF 212 kb) [file 12992_2016_223_MOESM2_ESM.pdf]

Additional file 2. Market share held by foreign firms in the grocery retail sector (%), 2004-2013, in selected Asian markets, with firm origin indicated

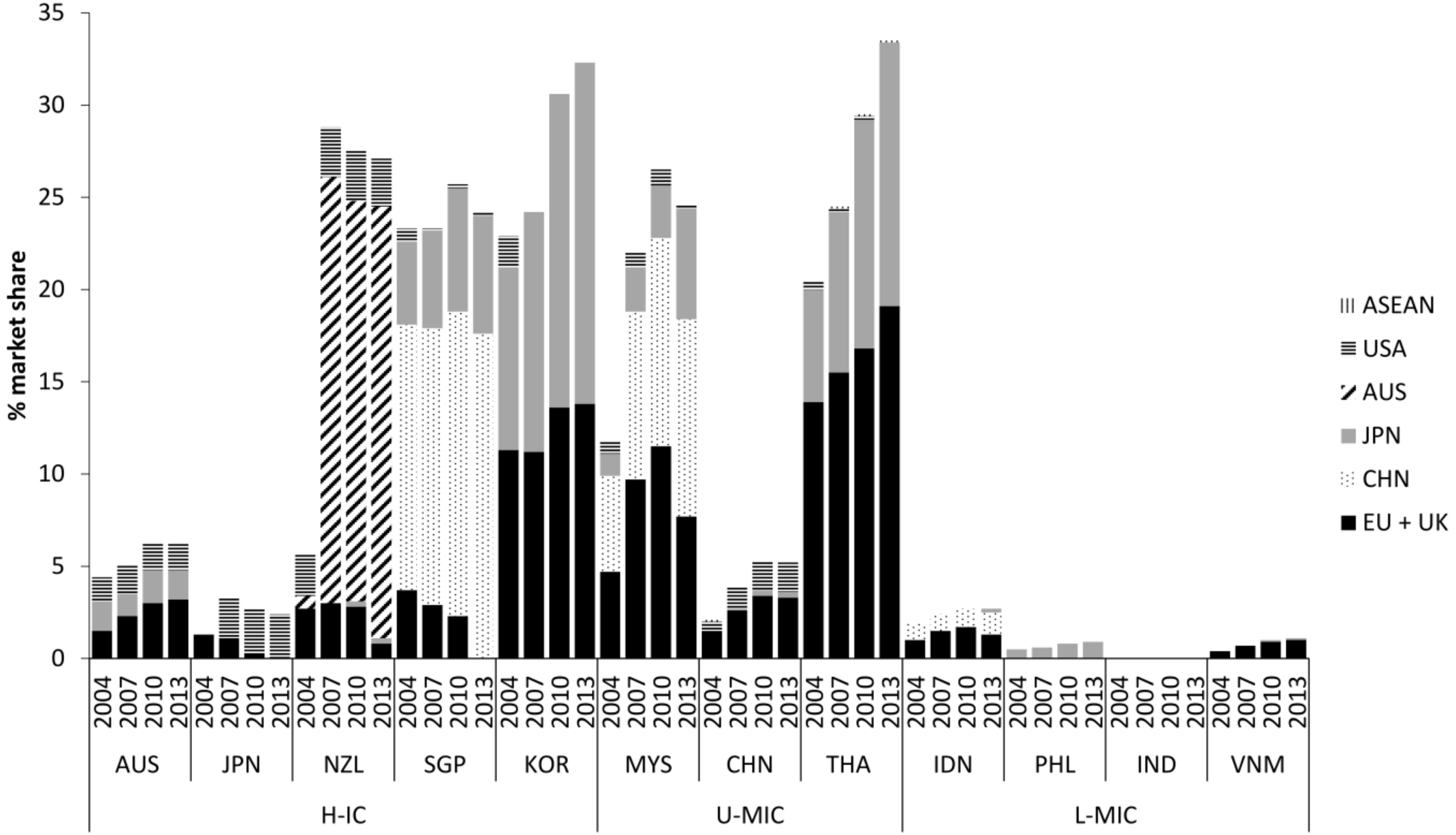

Footnotes: ASEAN = Association of South East Asian Nations; USA = United States of America; EU + UK = Switzerland and United Kingdom; H-IC = high-income countries; U-MIC = upper-middle income countries; L-MIC = lower-middle income countries; see methods section for other country abbreviations; data from [24].
