# Supplementary material for: Food systems transformations, ultra-processed food markets and the nutrition transition in Asia
Source: Global Health. 2016 Dec 3;12:80. doi: 10.1186/s12992-016-0223-3 (PMC5135831; doi:10.1186/s12992-016-0223-3)
Supplement: Additional file 3: — Sales transactions per capita at food service outlets, 1999–2013 with projections to 2017, in selected Asian markets (PDF 159 kb) [file 12992_2016_223_MOESM3_ESM.pdf]

Additional file 3. Sales transactions per capita at food service outlets, 1999-2013 with projections to 2017, in selected Asian markets

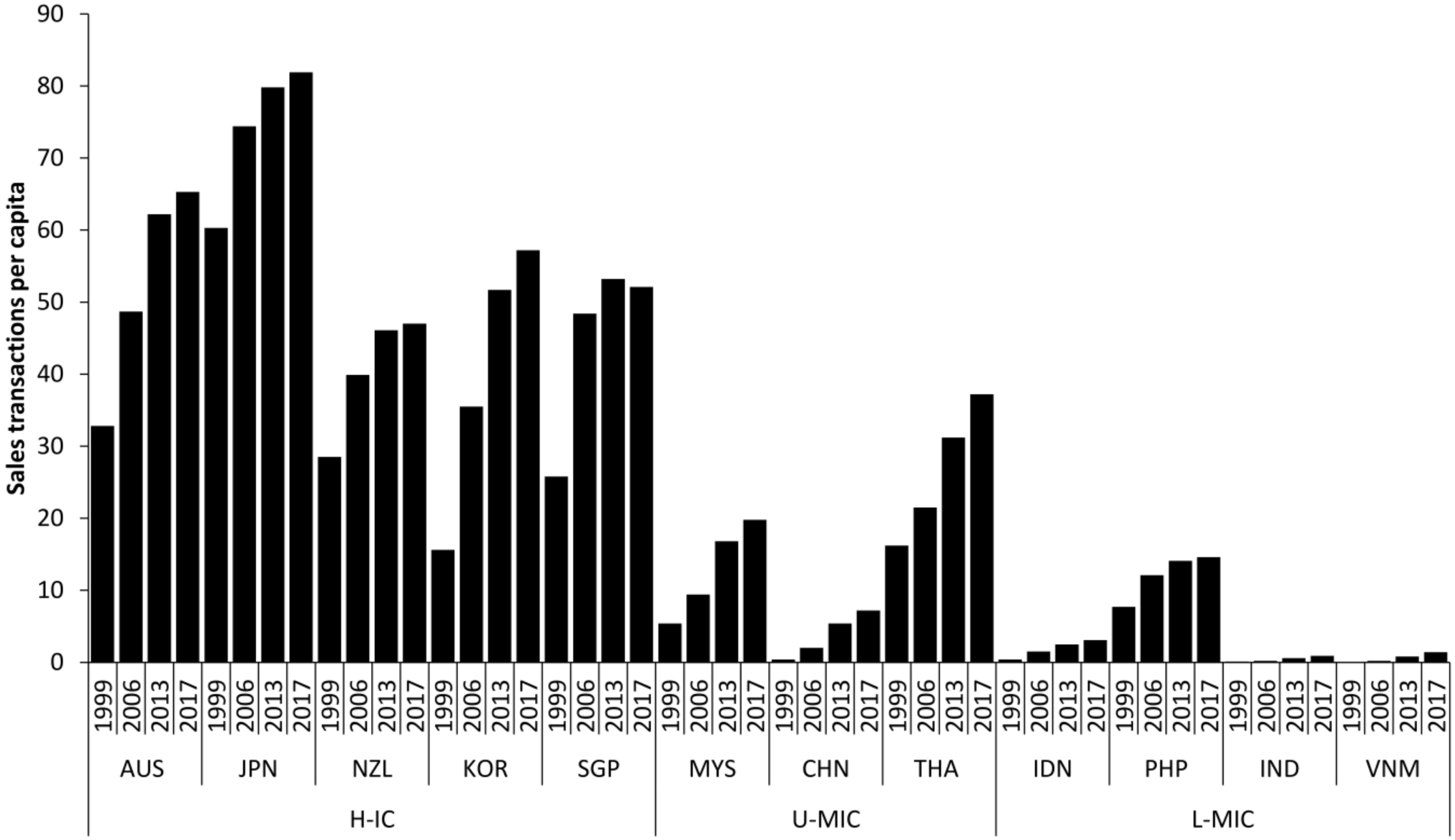

Footnotes: H-IC = high-income countries; U-MIC = upper-middle income countries; L-MIC = lower-middle income countries; see methods section for other country abbreviations; data from [24].
