# Supplementary material for: Food systems transformations, ultra-processed food markets and the nutrition transition in Asia
Source: Global Health. 2016 Dec 3;12:80. doi: 10.1186/s12992-016-0223-3 (PMC5135831; doi:10.1186/s12992-016-0223-3)
Supplement: Additional file 4: — Market concentration in the food service sector (% market share held by leading four firms), 2003–2012, in selected Asian markets, with company rank indicated (PDF 205 kb) [file 12992_2016_223_MOESM4_ESM.pdf]

Additional file 4. Market concentration in the food service sector (% market share held by leading four firms), 2003-2012, in selected Asian markets, with company rank indicated

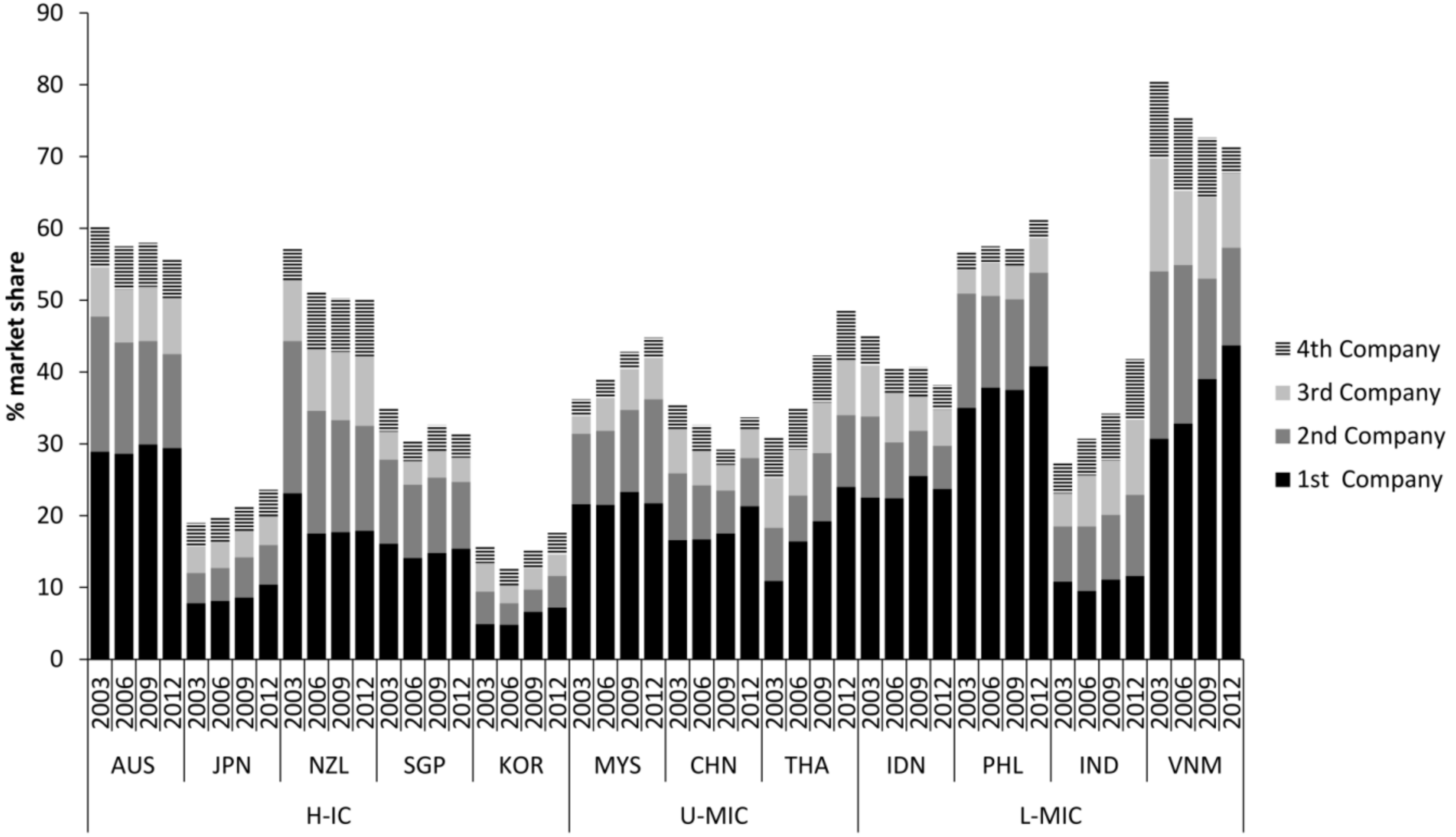

Footnotes: H-IC = high-income countries; U-MIC = upper-middle income countries; L-MIC = lower-middle income countries; see methods section for other country abbreviations; data from [24].
